# Scutellarin improves the radiosensitivity of non‐small cell lung cancer cells to iodine‐125 seeds via downregulating the AKT/mTOR pathway

**DOI:** 10.1111/1759-7714.14077

**Published:** 2021-07-13

**Authors:** Guang‐hui He, Dian‐jin Xing, Die Jin, Yue Lu, Lei Guo, Yu‐liang Li, Dong Li

**Affiliations:** ^1^ Cheeloo College of Medicine Shandong University Jinan China; ^2^ Department of Interventional Therapy The Second People's Hospital of Weifang Weifang China; ^3^ Department of Interventional Medicine The Second Hospital of Shandong University Jinan China; ^4^ Department of Vascular Anomalies and Interventional Radiology Qilu Children's Hospital of Shandong University Jinan China

**Keywords:** iodine radioactive seed, mTOR protein, non‐small cell lung cancer, Scutellarin

## Abstract

**Background:**

In our previous study, we indicated that scutellarin (SCU) induced an anticancer effect in A549 cells. However, whether SCU regulates the radiosensitivity of non‐small cell lung cancer (NSCLC) and its related mechanism is still unclear.

**Methods:**

In this study, we explored the anticancer effect induced by iodine‐125 (^125^I) and SCU at a sensitizing concentration in A549 and H1975 cells. Cellular apoptosis and proliferation were detected by flow cytometry, Bcl‐2/Bax expression level, cell cycle, CCK‐8, and EdU staining. A tumor model using nude mice was also carried out to investigate the combined effect of ^125^I and SCU in vivo. In addition, the expression level of AKT/mTOR pathway was detected to investigate whether it is linked to the anticancer effect of ^125^I and SCU.

**Results:**

SCU at a sensitizing concentration promoted the ^125^I‐induced apoptosis and antiproliferative effect in A549 and H1975 cells. Moreover, the same results were obtained in vivo. Based on our findings, the AKT/mTOR pathway was significantly downregulated after combined treatment with ^125^I and SCU.

**Conclusions:**

The results of our study suggested that SCU promotes the anticancer effects induced by ^125^I in NSCLC cells by downregulating the AKT/mTOR pathway and lays a foundation for future application of this combined treatment.

## Graphical abstract

Scutellarin (SCU) enhanced inhibition of ^125^I‐induced proliferation, promoted ^125^I‐induced apoptosis, and boosted ^125^I‐induced tumor inhibition in vivo in non‐small cell lung cancer (NSCLC) cells, as well as enhanced downregulation of the ^125^I‐induced AKT/mTOR pathway.

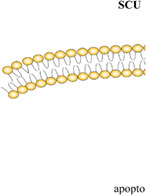

## INTRODUCTION

Non‐small cell lung cancer (NSCLC) is a type of lung cancer and one of the most common tumors worldwide. Currently, surgery combined with chemotherapy is the predominant treatment for NSCLC, which has a poor outcome and prognosis due to rapid development, drug resistance, and high recurrence and metastasis of lung cancer.^1^Therefore, there is an urgent need to elucidate the molecular mechanisms underlying lung cancer to lay a solid foundation for its diagnosis, prevention, and treatment. Radioactive iodine‐125 (^125^I) seed implants are a type of internal radiotherapy that can effectively kill tumor cells and protect the surrounding normal tissue, and are used as one of the treatments for advanced lung cancer.^2^ In addition, ^125^I combined with chemotherapy is considered a safe and effective method for NSCLC, but the exact mechanism remains unclear.

The development of new natural antitumor drugs has attracted attention worldwide, and it has been found that traditional Chinese medicine can effectively inhibit the growth of tumor cells, reduce the complications of tumors, and result in relatively few adverse effects.^3^ Scutellarin (SCU) is a flavonoid isolated from the stem and leaves of *Scutellaria baicalensis*.^4^ Recent studies have shown that SCU has an inhibitory effect on the growth of various tumors and is a potential antitumor drug for hepatocellular carcinoma,^5^ gastric, lung,^6^ prostate^7^ and colon cancers.^8^ SCU has been reported to regulate the proliferation of hepatocellular carcinoma cells by downregulating the levels of Bcl‐xl and Mcl‐1 in the STAT3 pathway.^9^ However, little is known about the antilung cancer mechanisms of SCU and whether it can sensitize the inhibitory effect induced by iodine radioactive seeds in NSCLC. In this study, we explored the antitumor effects of SCU, ^125^I, and their combined treatment on NSCLC in vivo and in vitro.

The AKT/mTOR pathway is a well‐studied anticancer pathway,^10^ and the PI3K/AKT/m TOR pathway has been found to be overactivated in liver cancer, cell survival, proliferation, migration, apoptosis, tumor metastasis, angiogenesis, and transformation.^11^ The AKT/mTOR pathway has been reported to inhibit NSCLC cell survival and induce cell autophagy and apoptosis in NSCLC.^12^ Several AKT/mTOR inhibitors have been evaluated for use in preclinical or clinical tumor therapy.

This study investigated the effects of SCU and ^125^I on the proliferation and apoptosis of human lung cancer A549 and H1975 cells, and whether SCU and ^125^I affect the biological behavior of NSCLC through the AKT/mTOR pathway. This study aimed to support our previous study and demonstrate the feasibility of SCU combined with ^125^I seeds as a strategy to improve the efficacy of NSCLC.

## MATERIALS AND METHODS

### In vivo tumor xenograft studies

A549 cells were injected subcutaneously into the hind legs of 4‐week‐old BALB/c male mice (Animal Research Center of Shandong University) at a concentration of 1 × 10^7^/ml. Tumor volume and body mass were measured every other day, and the maximum (L) and minimum (W) diameter of tumors in each group were measured with calipers and recorded. Tumor volume was calculated using the following formula: tumor volume = (L × W^2^)/2. After 30 days of continuous recording, nude mice were sacrificed using the cervical dislocation method, and subcutaneous graft tumors were peeled off and measured. In this study, mice were fed in specific pathogen‐free conditions and the experiment was approved by Shandong University Animal Care Committee followed by Ministry of Science and Technology of China.

### Radioactive ^125^I seed implants in mice

After the tumor volume reached 400 mm^3^, the tumors and surrounding skin were sterilized, and the seeds were inserted into the center of the tumors under anesthesia. ^125^I seeds and seed implant equipment were provided by the Ningbo Junan Pharmaceutical Technology Company.

### Cell culture

Human lung cancer cell lines A549 and H1975, purchased from the Chinese Academy of Sciences (Shanghai, China), were cultured in Dulbecco's modified Eagle medium (DMEM) containing a volume fraction of 10% fetal bovine serum (Gibco) and incubated at 37°C with 5% CO_2_.

### Sensitizing concentration of SCU

The cell suspensions were prepared by adjusting the cell density and were inoculated into 96‐well culture plates. According to the experiment, a concentration gradient ranging from 0 to 500 μg/ml was set up with five replicate wells for each concentration. After 72 h of incubation, the culture was terminated by adding 10 μl of CCK‐8 solution (Dojindo). Then, the culture was incubated again for 3 h. The absorbance was measured at 450 nm using a microplate reader (Thermo Fisher Scientific). Finally, the IC_50_ values of A549 and H1975 cells were calculated based on the collected values.

### Cell viability assay

After treatment, cells were prepared into cell suspensions for seeding into 96‐well plates with 200 μl suspension per well at a concentration of 5 × 10^3^ cells/well. After incubation for 24, 48, and 72 h, 10 μl of CCK‐8 was added and the cells were incubated again for 3 h. The optical density was measured at 450 nm, and cell survival rates were analyzed.

### Flow cytometry

The cells were collected after 72 h of treatment, washed twice with phosphate‐buffered saline (PBS) solution, and suspended in 500 μl of binding buffer solution. Propidium iodide (PI) and annexin V‐FITC (BD Biosciences) were added, and the mixture was incubated for 15 min at room temperature. Apoptotic changes were detected using a flow cytometer (BD Biosciences). Cells were fixed by adding 70% anhydrous ethanol solution after discarding the supernatant and stored at −20°C overnight to prepare for cell cycle analysis. Before detection, the cells were stained with PI and RNase A (Elabscience) for 30 min at room temperature.

### EdU assay

After the cells were treated as previously indicated, the culture medium was changed to EdU solution (RiboBio) and incubated for 2 h. Cells were stained with Apollo 567 and Hoechst 33342 for 30 min and observed under a laser confocal microscope (Olympus). Blue fluorescence indicated the nucleus and red fluorescence the EdU‐positive cells.

### Western blotting and antibodies

After treatment for 72 h, the cells were collected and washed twice with cold 1× PBS. Total protein was extracted using ultrasonic shaking at 12 000 rpm for 10 min, centrifugation, and supernatant collection. Separate gels of 100 g/l and concentrated gels of 0.05 volume fraction were prepared, and 10 μl of protein samples were added to each lane. The procedure was performed as described previously.^13^ The primary antibodies were purchased from Abcam and diluted as follows: p‐AKT (1:1000), p‐mTOR (1:1000), Bcl‐2 (1:1000), Bax (1:500), and GAPDH (1:1000).

### Statistical analyses

Graphpad Prism 6.0 software was used for data analysis, and the results were expressed as mean ± standard deviation (SD). The differences between groups were analyzed using one‐way analysis of variance and Tukey's multiple comparison test, and *p* < 0.05 was considered statistically significant.

## RESULTS

### **SCU enhanced inhibition of** ^**125**^ **I** **‐induced proliferation in NSCLC cells**

**FIGURE 1:**
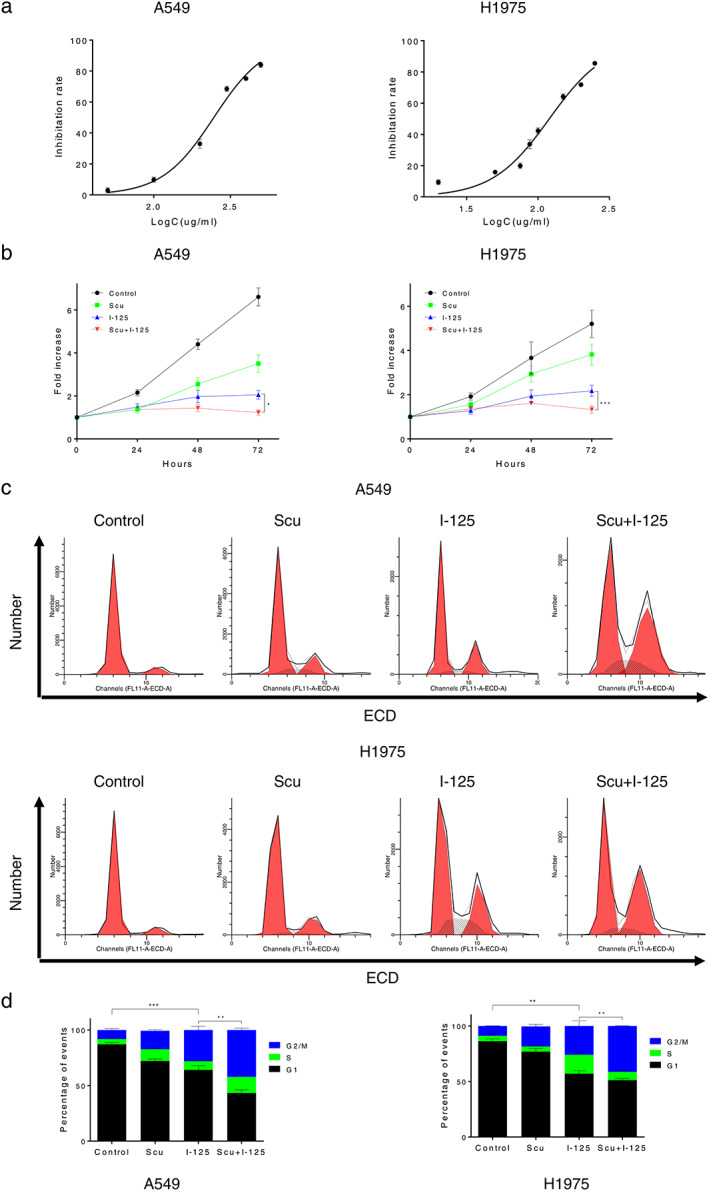
Scutellarin (SCU) enhanced ^125^I‐induced proliferation inhibition in NSCLC cells. (a) To investigate the half maximal inhibitory concentration (IC_50_) of SCU on A549 and H1975 cell lines, the concentration of SCU required for A549 and H1975 cell lines was determined. (b) To investigate the effect induced by ^125^I combined with SCU on antiproliferative cancer cells, proliferation was detected using a CCK‐8 assay. (c) A cell cycle assay was performed to verify the antiproliferative effects of SCU and ^125^I on A549 and H1975 cells. All experiments were performed in triplicate and the data are presented as the mean ± SD. *p < 0.05, **p < 0.01, ***p < 0.001

**FIGURE 2:**
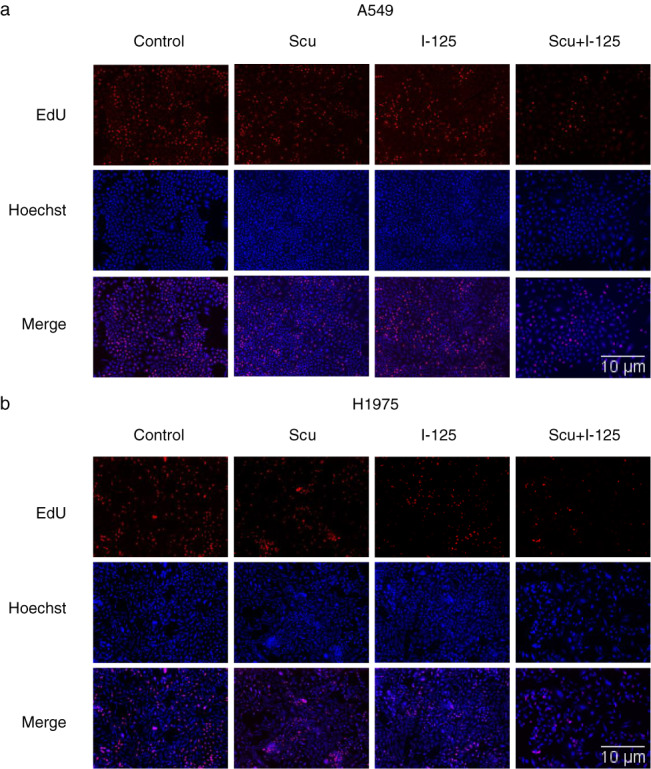
Scutellarin (SCU) enhanced ^125^I‐induced proliferation inhibition in NSCLC cells. (a,b) An EdU assay was carried out to evaluate the antiproliferative effects of SCU,^125^I, or ^125^I + SCU, on A549 and H1975. All experiments were performed in triplicate and the data are presented as the mean ± SD. *p < 0.05, **p < 0.01

First, we investigated the inhibition rate of a range of SCU in NSCLC cells to determine the sensitizing concentration of SCU, which was determined to be 10% of the IC_50_ (Figure 1(a)). The IC_50_ of SCU in A549 and H1975 cells after 72 h of treatment was 246.8 μg/ml and 120.0 μg/ml, respectively. After cells were treated with ^125^I, SCU, or both, cell proliferation was detected using a CCK‐8 assay. The inhibitory effect of the combined treatment on cell proliferation was stronger than that of single treatment (Figure 1(b)). Furthermore, the cell cycle assay suggested a G2/M arrest after the cells were treated with ^125^I, SCU, or both, which was more remarkable with combined treatment (Figure 1(c)). Moreover, the EdU assay showed a similar result: cell proliferation was more notably inhibited with combined treatment than with single treatment in A549 and H1975 cells (Figure 2).

### **SCU significantly promoted** ^**125**^ **I** **‐induced apoptosis in NSCLC cells**

**FIGURE 3:**
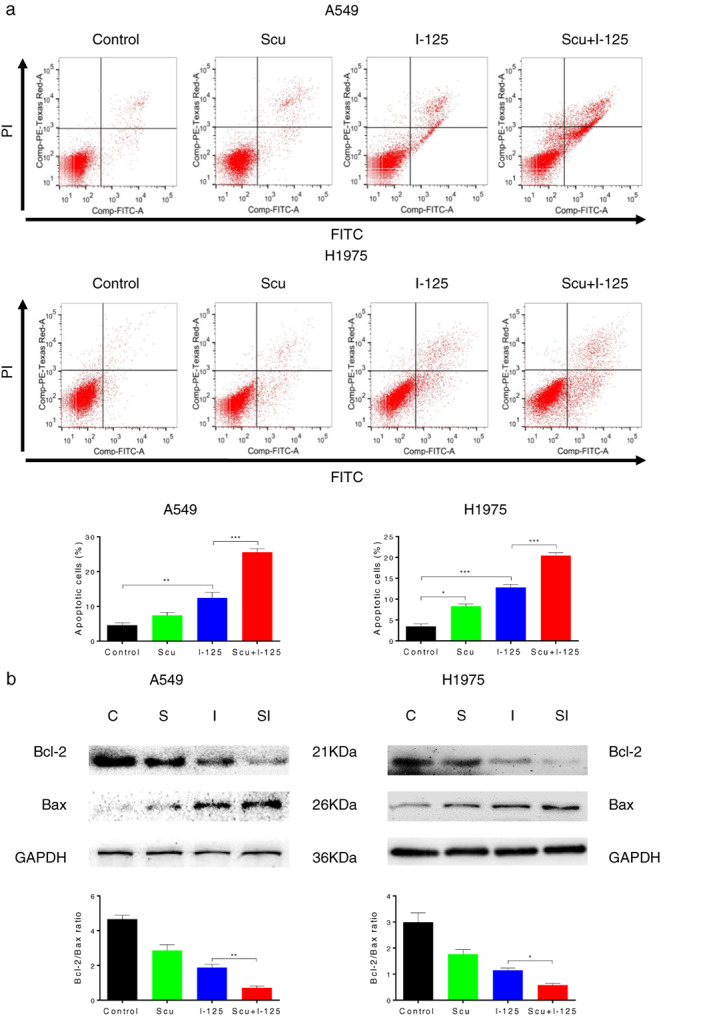
Scutellarin (SCU) significantly promoted ^125^I‐induced apoptosis in NSCLC cells. (a) After cells were treated with ^125^I, SCU, or ^125^I + SCU, annexin V/PI was performed to analyze cellular apoptosis. (b) Protein levels of Bcl‐2 and Bax regulated by ^125^I, SCU,^125^ and I + SCU in A549 and H1975 cells were detected by western blotting. Data are presented as the mean ± SD, *p < 0.05, **p < 0.01, ***p < 0.001

Cell apoptosis, detected using flow cytometry, was significantly increased after A549 and H1975 cells were treated with a combination of SCU and ^125^I (Figure 3(a)). Moreover, the expression level of Bcl2/Bax in A549 and H1975 cells, detected using western blotting, was higher with combined treatment than with single treatment (Figure 3(b)). In general, SCU significantly promoted ^125^I‐induced apoptosis in A549 and H1975 cells.

### **SCU boosted** ^**125**^ **I** **‐induced tumor inhibition in vivo**

**FIGURE 4:**
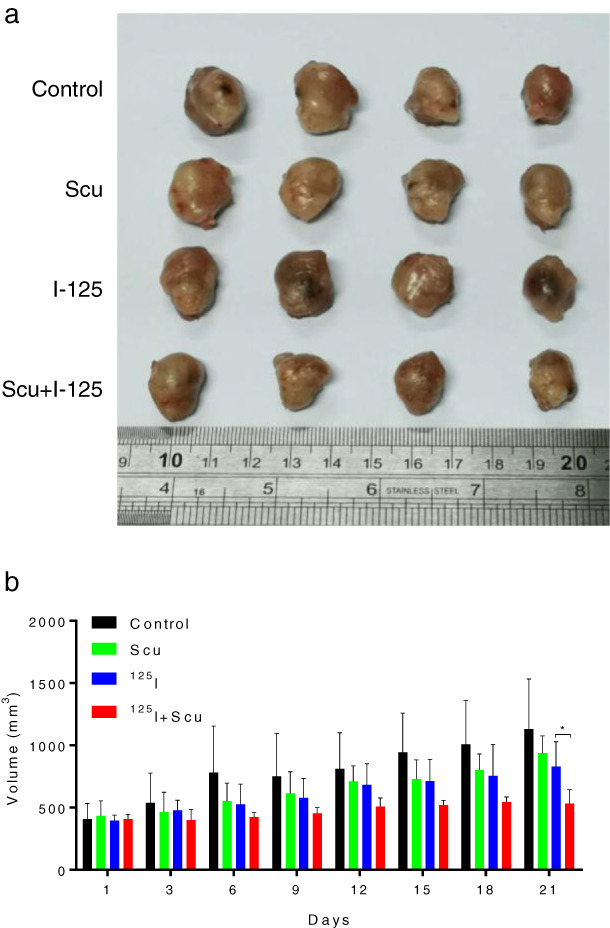
Scutellarin (SCU) boosted ^125^I‐induced tumor inhibition in vivo. (a,b) To further verify the effect of the ^125^I, SCU, ^125^I + SCU effect of apoptosis and antiproliferation in A549 and H1975 cells, a nude mice xenograft tumor assay was performed, and the mice were treated with ^125^I and SCU. The data are presented as the mean ± SD. *p < 0.05

To explore whether SCU could boost ^125^I‐induced tumor suppression in vivo, an A549 xenograft nude mouse model was used. After the mice were treated with SCU, ^125^I, or both, tumor growth was more obviously suppressed with combined treatment than with single treatment (Figure 4(a)). Furthermore, the tumor volume was remarkably reduced with combined treatment (Figure 4(b)). In general, SCU boosted ^125^I‐induced tumor inhibition in vivo.

### SCU downregulated the AKT/mTOR pathway

**FIGURE 5:**
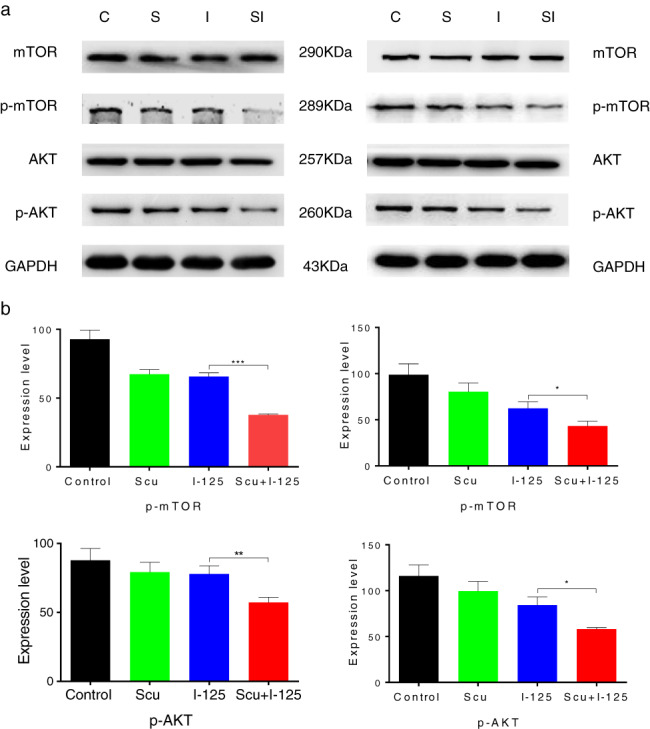
Scutellarin (SCU) enhanced the downregulation of the ^125^I‐induced AKT/mTOR pathway. (a,b) Expression levels of mTOR, p‐mTOR, AKT, p‐AKT in A549 and H1975 cells were detected after treatment with^125^I, SCU, and ^125^I + SCU. Data are presented as the mean ± SD, *p < 0.05, **p < 0.01, ***p < 0.001

To determine the molecular mechanism involved in SCU‐promoted ^125^I‐induced apoptosis and inhibition of proliferation in A549 and H1975 cells, the expression level of the AKT/mTOR pathway was detected after single or combined treatment. Western blotting showed that the AKT/mTOR pathway was more remarkably downregulated with combined treatment than with single treatment (Figure 5).

## DISCUSSION

^125^I radioactive seed implants have recently become an important therapeutic approach in the treatment of lung cancer. In our previous study, we found that ^125^I could induce apoptosis and inhibit proliferation in hepatocellular carcinoma and pancreatic cancer cells. However, the underlying mechanisms of ^125^I and whether ^125^I can inhibit NSCLC cells remain unclear. In this study, we explored whether SCU could enhance the radiosensitivity of NSCLC cells to ^125^I and the possible associations between ^125^I, SCU, and the AKT/mTOR pathway.

Modern pharmacological research shows that the active ingredient of wild SCU prevents ischemia–reperfusion injury of cardiomyocytes and is widely used in the treatment of cardiovascular and cerebrovascular diseases. However, many recent studies have shown that SCU can also have significant antitumor activities.^9^ Furthermore, SCU can sensitize oxaliplatin‐resistant colorectal cancer cells to oxaliplatin treatment through inhibition of PKM2.^14^ SCU may also suppress the differentiation of colon cancer stem cells by downregulating the activity of signaling pathways^15^ and can significantly reverse hypoxia‐induced epithelial to mesenchymal transition (EMT) in breast cancer (BC) cells, thus SCU could be a valuable anticancer agent in BC treatment.^16^ The combination of wild SCU and cisplatin (DDP) may improve the sensitivity of lung cancer A549/DDP cells to DDP and inhibit clone formation, and may be related to the downregulation of c‐met protein expression.^17^ Our previous study demonstrated that SCU can inhibit proliferation of A549 cells and promote apoptosis through the AKT/mTOR/4EBP1 and STAT3 pathways.^18^ In this study, we found that SCU could enhance the radiosensitivity of NSCLC cells to ^125^I by downregulating the AKT/mTOR pathway.

In recent years, ^125^I seeds have been regarded as an effective treatment for NSCLC because of their low radioactivity, high accuracy, low trauma to surrounding normal tissues, and high conformability.^19^, ^20^ However, the mechanism of ^125^I in combination with chemotherapy in NSCLC is unclear; ^125^I has been reported to inhibit tumor growth in A549 xenograft tumors by suppressing the Warburg effect.^21^ A recent study on lung adenocarcinoma showed that radioactive ^125^I seed implantation therapy inhibited proliferation of tumor cells, retarded tumor growth, and promoted apoptosis in human lung adenocarcinoma A549 cells by upregulating p21 and Caspase‐9 protein expression, and downregulating survivin and livin protein expression.^22^ In the present study, we investigated the radiobiological effects of ^125^I seeds on NSCLC cells and demonstrated that ^125^I significantly inhibited tumor growth in vivo and in vitro, which was enhanced by SCU.

The AKT/mTOR pathway is a popular pathway that relates cell apoptosis, proliferation, and cell differentiation. A recent study revealed that ginkgolic acid can affect the EMT of lung cancer cells by downregulating the AKT/mTOR pathway, thereby inhibiting migration and metastasis.^23^ In addition, rapamycin with AKT inhibitors can alter the AKT/mTOR pathway to regulate EMT, stemness, and metastasis in nasopharyngeal carcinoma cells.^24^ Furthermore, resveratrol exerts anticancer effects in oral cancer by inhibiting the PI3K/AKT pathway, thereby significantly enhancing the sensitivity of lipopolysaccharide‐binding proteins.^25^ Our results show that drugs that target the AKT/mTOR signaling pathway should be new candidates for radiosensitization.^26^ A large body of preclinical evidence suggests that AKT/mTOR pathway inhibitors can be effectively used in combination with chemotherapy, radiotherapy, and targeted agents to improve efficacy and overcome resistance, suggesting that the mTOR pathway may be a target for promoting tumor sensitivity to radiotherapy.^27^ In this study, we found that SCU enhanced the downregulation of the ^125^I‐induced AKT/mTOR pathway, suggesting that AKT/mTOR might be a potential target to regulate radiosensitivity.

The data in this study suggest that SCU enhanced the anticancer effect of ^125^I through regulating the AKT/mTOR pathway. Although our study gained positive results, there are still some limitations. First, the potential mechanism should be investigated in detail. And more NSCLC cell lines should be used to better improve the conclusion. In addition, AKT/mTOR pathway had been investigated in many studies, further research should be carried outin the future.

## CONFLICT OF INTEREST

There is no conflict of interest in this manuscript.
